# Rates of transposition in *Escherichia coli*

**DOI:** 10.1098/rsbl.2013.0838

**Published:** 2013-12-23

**Authors:** Ana Sousa, Catarina Bourgard, Lindi M. Wahl, Isabel Gordo

**Affiliations:** 1 Instituto Gulbenkian de Ciência, Oeiras, Portugal; 2 Department of Applied Mathematics, Western University, London, Ontario, Canada

**Keywords:** transposable elements, insertion sequences, rate of transposition, *Escherichia coli*, mutation accumulation

## Abstract

The evolutionary role of transposable elements (TEs) is still highly controversial. Two key parameters, the transposition rate (*u* and *w*, for replicative and non-replicative transposition) and the excision rate (*e*) are fundamental to understanding their evolution and maintenance in populations. We have estimated *u*, *w* and *e* for six families of TEs (including eight members: IS1, IS2, IS3, IS4, IS5, IS30, IS150 and IS186) in *Escherichia coli*, using a mutation accumulation (MA) experiment. In this experiment, mutations accumulate essentially at the rate at which they appear, during a period of 80 500 (1610 generations × 50 lines) generations, and spontaneous transposition events can be detected. This differs from other experiments in which insertions accumulated under strong selective pressure or over a limited genomic target. We therefore provide new estimates for the spontaneous rates of transposition and excision in *E. coli*. We observed 25 transposition and three excision events in 50 MA lines, leading to overall rate estimates of *u* ∼ 1.15 × 10^–5^, *w* ∼ 4 × 10^−8^ and *e* ∼ 1.08 × 10^−6^ (per element, per generation). Furthermore, extensive variation between elements was found, consistent with previous knowledge of the mechanisms and regulation of transposition for the different elements.

## 1. Introduction

Transposable elements (TEs) are thought to be major players in genome organization and evolution. In prokaryotes, the simplest autonomous TEs are called insertion sequences (ISs). Usually, these elements encode no other function than their own mobility: a transposase flanked by short inverted repeats. IS elements are very common in bacterial genomes [1] and show an outstanding ability to mobilize within the host genome [2]. The outcome of such invasion is sometimes unexpected, with cases where transposition was responsible for direct fitness increases [3] or played a major role in adaptation to specific environments [4]. However, most spontaneous transposition events lead to a reduction in the growth rate of bacteria [5]. Consistent with the selfish DNA hypothesis, TEs can nevertheless be maintained because of their high transposition rate despite the deleterious effects they may cause. Theory [6] suggests that, in prokaryotes, TEs can be maintained if there is horizontal transfer and the rate of transposition and excision decreases with copy number [7]. Knowledge about transposition and excision rates is therefore crucial for understanding TE abundance in natural populations [8].

Estimates of the transposition and excision rates of IS elements vary widely, ranging from 10^−3^ to 10^−7^ for transposition [9,10] and about 10^−9^ to 10^−10^ for excision [11,12]. These estimates were typically obtained by ‘mating-out’ assays [9], papillation assays [13] and fluctuation tests. Estimates obtained by direct observation are rare [14]. Notably, all approaches except direct observation manipulate the system in order to detect events that are very infrequent.

Here, we estimate the spontaneous rates of copy-and-paste (*u*), cut-and-paste (*w*) and excision (*e*) per element, per generation for eight different IS elements, using mutation accumulation (MA) in a mutator strain of *Escherichia coli*. In our experiment, natural selection is kept at a minimum long enough that a significant number of transposition events, even if deleterious, accumulate. This approach specifically aims at an unbiased estimation of the transposition rate, excluding lethal events.

## 2. Material and methods

### (a) Bacterial strains and growth conditions

The strain used in this study was *E. coli* K12 MG1655 *srl*::Tn10 *mutS* (ancestor of the MA). The 50 lines for which we determined new transposition events were derived from a previous MA experiment which we prolonged for an extra 20 passages following the same protocol. All cultures were grown on Luria–Bertani medium supplemented with agar at 37°C.

### (b) DNA extraction and detection of transposition events

DNA isolation from the 50 MA lines at the 70th passage, as well as from the ancestor, was done following a previously described protocol [15]. To determine the number of IS copies in the genome of the ancestor and in the MA lines, we used the vectorette PCR (vPCR) method described in [16]. To reduce the probability of missing transposition events, we performed the vPCR method with two different enzymes (RsaI and BstUI). As shown in [16], this procedure allows a reliable determination of the number of copies of ISs in *E. coli*'s genome. However, because we used a mutator strain, all events were individually confirmed by target sequencing (see the electronic supplementary material for details). The vPCR method revealed 41 events of transposition (including excisions), from which 28 events were confirmed. Half of the non-confirmed events were due to another IS element (IS10), which was present in the ancestor, but was not targeted in this study (its transposition rate approx. 10^−4^ [14] is too high to be accurately estimated by this methodology). Insertions of IS10, either close to or within the targeted ISs, largely explain the mismatch. One third of the non-confirmed events (excisions) were caused by the deletion of a DNA fragment close to the element but not the element itself. Finally, the remainder of the non-confirmed events resulted from point mutations that presumably affected the restriction site or annealing of a primer.

### (c) Estimating the rate of excision and transposition

We used a multi-type branching process [17] to estimate the rates *u*, *w* and *e* independently for each IS. Briefly, we model types (*a*,*b*,*c*) in which *a* copy-and-paste events, *b* excisions and *c* cut-and-paste events have occurred, with respect to the ancestral genotype. Type (*a*,*b*,*c*) thus carries *m* = *m*_0_ + *a − b* copies of the IS, where *m*_0_ is the ancestral copy number for that IS. We assume that type (*a*,*b*,*c*) produces an individual in the next generation of type (*a* + 1,*b*,*c*), type (*a*,*b* + 1,*c*) or type (*a*,*b*,*c* + 1) with probabilities m*u*, m*e* and m*w*, respectively; the probability of double events per generation is neglected because the rates are expected to be low. For particular values of *u*, *w* and *e*, we constructed a Markov chain to describe this process over 70 passages, recovering a predicted distribution of types. We then used nonlinear minimization (Nelder–Mead) to find values of *u*, w and *e* that minimize the sum of squared error between observed and predicted frequencies of each type.

## 3. Results

### (a) Accumulation of spontaneous transpositions

**Figure 1.**
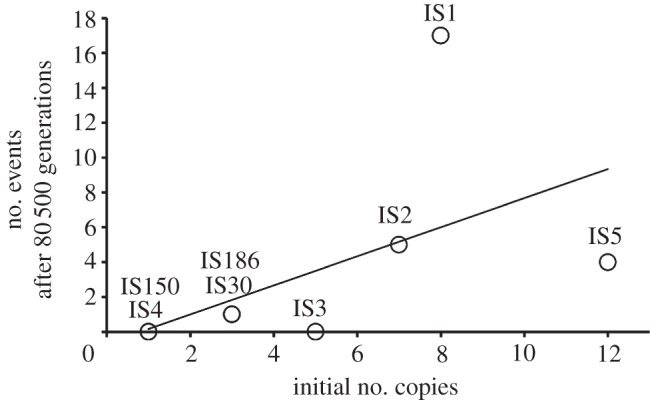
Number of transposition events as a function of initial copy number (for a given IS element). The regression equation is *y* = 0.83*x* – 0.67, *r*^2^ = 0.30, *p* = 0.04.

We determined the transposition and excision events that occurred in 50 MA lines (table 1). In 58% of the lines no events were detected, the remaining lines exhibited between one and three events and more than one event involving the same IS type was detected in only two lines. We observed a significant correlation between the number of initial copies of each IS type and the number of events observed after 80 thinsp;500 generations (figure 1; Spearman's coefficient = 0.78, *p* = 0.022). This is expected under the conditions used in this study where selection is minimized and the number of observed events should reflect the spontaneous rate of transposition and be proportional to the initial number of copies. It is commonly thought that many TEs are auto-regulated, i.e. their rate of transposition decreases as the copy number increases [10]. Our data do not lend support to this expectation (see electronic supplementary material, figure S1), though the sample size was not sufficient to make definitive conclusions.

**Table 1.** Transpositions events confirmed by sequencing per line (from the 70th passage) per IS element type. In the first line, the copy number of each IS element in the reference genome (NC_000913) and in the ancestor is indicated. Each new event is represented as follows: copy-and-paste (+1), cut-and-paste (+1−1) and excision (−1). 29 lines registered no detectable new events. ISs 4, 30 and 150 are not in the table because no transposition events were detected for these elements.

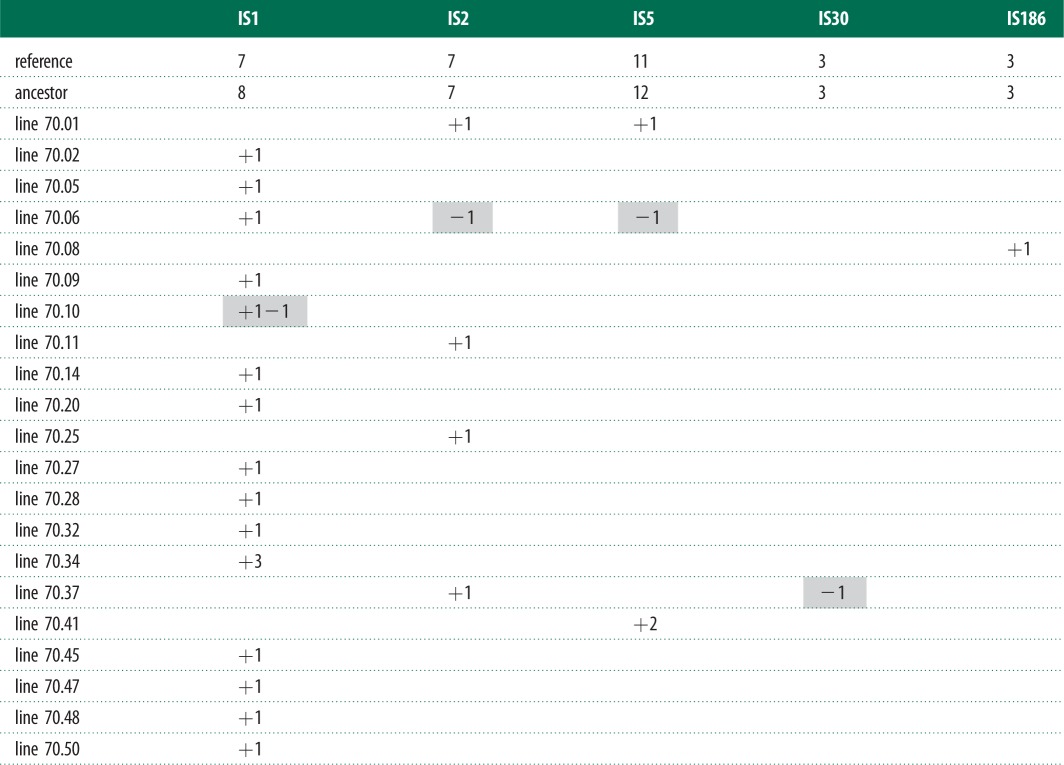

### (b) Inferring the ‘effective’ rates of transposition and excision

We estimate the ‘effective’ rates of transposition and excision, that is, the end result of the spontaneous process in terms of keeping, increasing or decreasing the IS copy number. For example, we score all events of IS loss as excisions (or cuts), irrespective of whether they occurred through the classic excision mechanism or were the result of deletions involving a particular IS. Among the IS elements studied, the ‘effective’ preferential mode of transposition is by far the copy-and-paste mechanism. Another clear result is the excess of events including IS1, which represent about 60% of all events. The highest rate of transposition (*u* = 2.67 × 10^−5^ ± 7.89 × 10^−6^) was estimated for IS1, followed by IS2 (*u* = 7.72 × 10^−6^ ± 3.29 × 10^−6^). Overall, *u* varied between 2.67 × 10^−5^ and 1.38 × 10^−6^ ± 1.18 × 10^−6^ (IS5). When pooling all the IS elements present in this strain of *E. coli*, excision events were rare: *e* estimates are 10 times lower than *u*. This implies that a reduction in IS copy number by spontaneous mutation alone is unlikely. However, we also found that for some elements (IS2 and IS5), *e* can be almost as high as *u*.

Over the 80 500 generations, no events were detected for IS3, IS4 and IS150. In the case of IS4 and IS150, the elements were present in the ancestral genome as a single copy; thus, even if they were to transpose at a rate as high as 10^−5^, for example, we would need more than 100 000 generations to detect one event (a timescale larger than in our experiment). As for IS3, it is possible that its transposition rate is in fact lower than that of all the others, because all five copies of this element were functional in the ancestral genome, as determined by sequencing.

## 4. Discussion

Previous estimates of transposition rates in bacteria span several orders of magnitude, from 10^−3^ to 10^−7^ [9,10]. Here, we estimate a range of *u* and *w* from 2.67 × 10^−5^ to 1.16 × 10^−6^. Our estimates are less biased than previous estimates, because selection was minimized and transposition was assessed across the whole genome. Furthermore, we include a sufficient number of different IS elements to account for differences between transposition modes.

**Figure 2.**
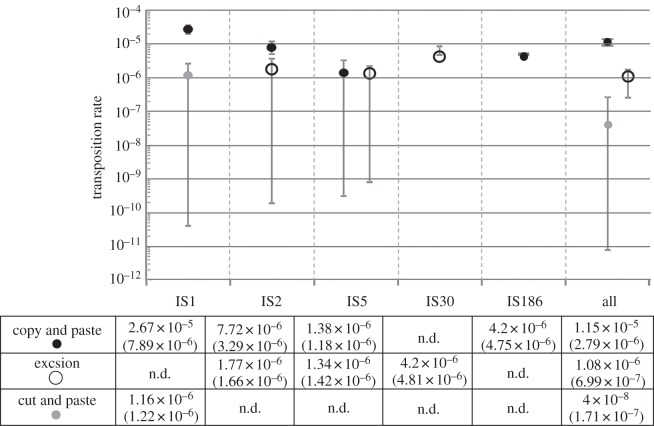
Estimates of transposition rates per element, per generation. Standard deviations are given between brackets. These estimates are based on data reported in table 1. See §2 for details of the analysis. Black and grey dots represent *u* and *w*, respectively*.* Open circles represent rates of excision. n.d. means non determined. Please note that the minimum estimate of *u* for IS186 and *e* for IS30 cover zero, therefore these cannot be represented in a logarithmic scale.

It has been reported that the spontaneous transposition of IS1 occurs at the relatively low rate of about 10^−7^ in a standard mating-out assay [18]. These assays involve both conjugation and transposition and are therefore likely to underestimate the transposition rate. Other strategies have also been used. Saito *et al.* [13] used a papillation assay to estimate the transposition rate of IS1 from a donor plasmid to the chromosome. Briefly, this assay scores a transposition of the IS element (which carries a gene lacking transcription and translational start signals) when it inserts in some position of the genome that restores the gene's expression. This approach only scores as transposition events the ones that restore the gene's function; moreover, it employs a greatly increased expression level of the transposase in order to observe such events. For these reasons, the estimated transposition rate of the order of 10^−3^ obtained with this method is difficult to interpret. Our estimates for this element, about 10^−5^ for *u* and 10^−6^ for *w*, somewhat reconcile these extremely disparate previous estimates. Conditions such as non-optimal temperature [19], starvation [20] and other potential stressors can also affect the rate and spectrum of transposition. For example, contrary to our observations (figure 2), Naas *et al*. [20] detected a rate of transposition for IS5 higher than that for IS1 during storage. Our estimates relate to a period during which *E. coli* is actively growing (daily transfers), which may explain the differences in the estimates.

The estimated rate of transposition averaged for all the elements of *E. coli* studied (*u* ∼ 1.15×10^−5^) is higher than the average excision rate (approx. 1.08×10^−6^), which suggests that in addition to excision, selection against new insertions helps keeping element numbers low.

A similar procedure from an MA experiment in *Drosophila melanogaster* produced mean estimates of *u* and *e* of 1×10^−4^ and 4 × 10^−6^ [21]. Understanding why these are about one order of magnitude higher than those in *E. coli* is an important question, which might be related to the remarkable difference in genome complexity between *E. coli* and *D. melanogaster*.

## References

[1] Mahillon J Chandler M 1998 Insertion sequences. Microbiol. Mol. Biol. Rev. 62, 725–774 9729608 10.1128/mmbr.62.3.725-774.1998 PMC98933

[2] Parkhill J 2003 Comparative analysis of the genome sequences of *Bordetella pertussis*, *Bordetella parapertussis* and *Bordetella bronchiseptica*. Nat. Genet. 35, 32–40 (doi:10.1038/ng1227) 12910271 10.1038/ng1227

[3] Hartl DL Dykhuizen DE Miller RD Green L de Framond J 1983 Transposable element IS50 improves growth rate of *E. coli* cells without transposition. Cell 35, 503–510 (doi:10.1016/0092-8674(83)90184-8) 6317194 10.1016/0092-8674(83)90184-8

[4] Casacuberta E González J 2013 The impact of transposable elements in environmental adaptation. Mol. Ecol. 22, 1503–1517 (doi:10.1111/mec.12170) 23293987 10.1111/mec.12170

[5] Elena SF Ekunwe L Hajela N Oden SA Lenski RE 1998 Distribution of fitness effects caused by random insertion mutations in *Escherichia coli*. Genetica 102–103, 349–358 (doi:10.1023/A:1017031008316) 9720287

[6] Sawyer SA Dykhuizen DE DuBose RF Green L Mutangadura-Mhlanga T Wolczyk DF Hartl DL 1987 Distribution and abundance of insertion sequences among natural isolates of *Escherichia coli*. Genetics 115, 51–63 3030884 10.1093/genetics/115.1.51 PMC1203063

[7] Bichsel M Barbour AD Wagner A 2010 The early phase of a bacterial insertion sequence infection. Theor. Popul. Biol. 78, 278–288 (doi:10.1016/j.tpb.2010.08.003) 20816882 10.1016/j.tpb.2010.08.003

[8] Touchon M Rocha EPC 2007 Causes of insertion sequences abundance in prokaryotic genomes. Mol. Biol. Evol. 24, 969–981 (doi:10.1093/molbev/msm014) 17251179 10.1093/molbev/msm014

[9] Craig NL 1996 Transposition. In EcoSal—Escherichia coli and Salmonella: cellular and molecular biology (ed. Neidhardt F), pp. 2239–2362 Washington, DC: ASM Press

[10] Craig NL, American Society for Microbiology 2002 Mobile DNA II. Washington, DC: ASM Press

[11] Kleckner N 1989 Transposon Tn10. In Mobile DNA (eds Berg DE Howe MM), pp. 227–268 Washington, DC: ASM Press

[12] Berg DE Egner C Hirschel BJ Howard J Johnsrud L Jorgensen RA Tlsty TD 1981 Insertion, excision, and inversion of Tn5. Cold Spring Harb. Symp. Quant. Biol. 45, 115–123 (doi:10.1101/SQB.1981.045.01.020) 6271454 10.1101/sqb.1981.045.01.020

[13] Saito T Chibazakura T Takahashi K Yoshikawa H Sekine Y 2010 Measurements of transposition frequency of insertion sequence IS1 by GFP hop-on assay. J. Gen. Appl. Microbiol. 56, 187–192 (doi:10.2323/jgam.56.187) 20647675 10.2323/jgam.56.187

[14] Shen MM Raleigh EA Kleckner N 1987 Physical analysis of Tn10- and IS10-promoted transpositions and rearrangements. Genetics 116, 359–369 3038673 10.1093/genetics/116.3.359 PMC1203147

[15] Wilson K 2001 Preparation of genomic DNA from bacteria. In Current protocols in molecular biology (eds Ausubel FM Brent R Kingston RE Moore DD Seidman JG Smith JA Struhl K), pp. 00:2.4.1–2.4.5 Hoboken, NJ: John Wiley & Sons, Inc 10.1002/0471142727.mb0204s56 18265184

[16] Zhong S Dean A 2004 Rapid identification and mapping of insertion sequences in *Escherichia coli* genomes using vectorette PCR. BMC Microbiol. 4, 26 (doi:10.1186/1471-2180-4-26) 15242519 10.1186/1471-2180-4-26 PMC481064

[17] Linda J Allan S 2003 An introduction to stochastic processes with applications to biology. Upper Saddle River, NJ: Pearson Prentice Hall

[18] Siguier P Perochon J Lestrade L Mahillon J Chandler M 2006 ISfinder: the reference centre for bacterial insertion sequences. Nucleic Acids Res. 34, D32–D36 (doi:10.1093/nar/gkj014) 16381877 10.1093/nar/gkj014 PMC1347377

[19] Ohtsubo Y Genka H Komatsu H Nagata Y Tsuda M 2005 High-temperature-induced transposition of insertion elements in *Burkholderia multivorans* ATCC 17616. Appl. Environ. Microbiol. 71, 1822–1828 (doi:10.1128/AEM.71.4.1822-1828.2005) 15812007 10.1128/AEM.71.4.1822-1828.2005 PMC1082539

[20] Naas T Blot M Fitch WM Arber W 1994 Insertion sequence-related genetic variation in resting *Escherichia coli* K-12. Genetics 136, 721–730 7911771 10.1093/genetics/136.3.721 PMC1205879

[21] Maside X Assimacopoulos S Charlesworth B 2000 Rates of movement of transposable elements on the second chromosome of *Drosophila melanogaster*. Genet. Res. 75, 275–284 (doi:10.1017/S0016672399004474) 10893864 10.1017/s0016672399004474

